# Supplementary material for: Reversible On/Off Switching of Ferroelectricity in a Molecular FeCo Prussian Blue Analogue with Multiple Control
Source: Nat Commun. 2026 Mar 7;17:3609. doi: 10.1038/s41467-026-70427-x (PMC13096190; doi:10.1038/s41467-026-70427-x)
Supplement: Supplementary file 1 — Supplementary Information [file 41467_2026_70427_MOESM1_ESM.pdf]

Supplementary Information for

Reversible On/Off Switching of Ferroelectricity in a Molecular FeCo Prussian Blue Analogue with Multiple Control

Yu-Bo Huang[1,2], Sheng-Qun Su[1,3]*, Wen-Huang Xu[1,4], Wen-Wei Zheng[1], Tian-Chi Ji[1,5], Kai-Ge Gao[6], Hui-Hui Cui[7], Shimon Ikenaga[8], Kaoru Yamamoto[9], Zhao-Yang Li[10], Shu-Qi Wu[1]* & Osamu Sato[1]*

[1]Institute for Materials Chemistry and Engineering and IRCCS, Kyushu University, 744 Motooka, Nishi-ku, Fukuoka, 819-0395, Japan

[2]Institute for Solid State Physics, The University of Tokyo, Kashiwa, Chiba, 277-8581, Japan

[3]Department of Chemistry, School of Science, Institute of Science Tokyo, 2-12-1, O-okayama, Meguro-ku, Tokyo 152-8551, Japan

[4]State Key Laboratory of Optical Fiber and Cable Manufacture Technology, Yangtze Optical Fibre and Cable joint stock limited company; Optics Valley Laboratory, Hubei 430073, PR China

[5]Shenyang National Laboratory for Materials Science, Institute of Metal Research, Chinese Academy of Sciences, 72 Wenhua Road, Shenyang, Liaoning, PR China

[6]College of Physical Science and Technology, Yangzhou University, Jiangsu 225009, PR China

[7]School of Chemistry and Chemical Engineering, Nantong University, Nantong 226019, PR China

[8]Graduate School of Science and Engineering, Okayama University of science, 1-1 Ridaicho, Okayama, 700-0005, Japan

[9]Department of Physics, Faculty of Science, Okayama University of Science, 1-1 Ridaicho, Okayama, 700-0005, Japan

[10]School of Materials Science and Engineering, Nankai University, Tianjin 300350, PR China

This PDF file includes:

Figure S1: Molecular structure and powder X-ray diffraction (XRD) analysis.

Figure S2: Temperature-dependent infrared spectra of **1**.

Figure S3: Temperature-dependent UV/vis absorption spectra of **1**.

Figure S4: Molecular structure, spin and valence states of metal centers, and space group switching of **1**.

Figure S5: Thermalgravimetric analyses (TGA) of **1** and **1’** under air flow.

Figure S6: Temperature-dependent infrared spectra of **1’**.

Figure S7. Molecular structure, space group, and spin and valence states of the metal centers between**1** and **1’** during dynamic vapor sorption.

Figure S8: Molecular stacking and space group of **1** at LT, MS* and HT phase.

Figure S9. Temperature-dependence of pyroelectric coefficient (*p*) based on single-crystal sample of **1**.

Figure S10. Temperature-dependent polarization changes based on powder compacts of **1**.

Figure S11. Raw data of pyroelectric measurements based on single-crystal sample of **1** after irradiation with 785 nm light in a sweep rate of 10 K min^{-1}.

Table S1: Crystallographic parameters of complex **1** at 100 K (LT phase), and 300 K (HT phase).

Table S2: Characteristic bond lengths (Å) of complex **1** at 100 K (LT phase), 300 K (HT phase).

Table S3: Crystallographic parameters of complex **1** at 30 K (MS* phase), and 30 K (MS phase).

Table S4: Characteristic bond lengths (Å) of complex **1** at 30 K (MS* phase), and 30 K (MS phase).

Table S5: Crystallographic parameters of complex **1’** at 120 K (LT phase), and 300 K (HT phase).

Table S6: Characteristic bond lengths (Å) of complex **1’** at 120 K (LT phase), 300 K (HT phase).

Point Charge Calculation. According to the 100 K crystal structure data, we select a unit cell and assume that the center of the positive charge locates on Fe and Co atoms, negative charges on C, N and B atoms, respectively.

Atom	Center coordination
	100 K
Fe^{2+}	(6.195379, 5.219564, 14.386917)
	(17.220114, 6.301636, 3.196098)
	(16.756698, 10.610795, 14.348868)
	(7.271134, 0.910405, 3.158049)
Fe^{3+}	(2.701857, 4.997551, 8.073057)
	(13.726592, 6.523649, -3.117762)
	(12.841941, 10.445581, 8.135726)
	(3.356377, 1.075619, -3.055094)
Co	(4.582679, 1.850766, 11.204248)
	(15.607415, 9.670434, 0.013429)
	(14.915461, 7.294648, 11.208725)
	(5.429896, 4.226552, 0.017905)
C	(6.570167, 6.23873, 12.784392)
	(3.672566, 3.837712, 9.138423)
	(7.735155, 4.16837, 13.977333)
	(1.173413, 3.921816, 8.466974)
	(5.323788, 3.960989, 13.252168)
	(2.340557, 6.039413, 9.628581)
	(17.594902, 5.28247, 1.593573)
	(14.697302, 7.683488, -2.052396)
	(18.75989, 7.35283, 2.786514)
	(12.198149, 7.599384, -2.723845)
	(16.348524, 7.560211, 2.061349)
	(13.365292, 5.481787, -1.562238)

	(17.075338, 11.672128, 12.757534)<br>(18.330934, 9.575269, 13.885569)<br>(15.792349, 9.411668, 13.220834)<br>(13.80173, 9.228481, 9.180948)<br>(12.574577, 11.470507, 9.72706)<br>(11.24561, 9.464666, 8.502784)<br>(7.589774, -0.150928, 1.566715)<br>(8.845369, 1.945931, 2.694749)<br>(6.306784, 2.109532, 2.030015)<br>(4.316166, 2.292719, -2.009871)<br>(3.089012, 0.050693, -1.463759)<br>(1.760045, 2.056534, -2.688035)
N	(4.937865, 3.197133, 12.511336)<br>(6.845094, 6.788291, 11.828696)<br>(8.593778, 3.45636, 13.719944)<br>(4.226366, 3.123397, 9.883732)<br>(2.128133, 6.654645, 10.599944)<br>(0.282436, 3.243218, 8.717648)<br>(15.962601, 8.324067, 1.320517)<br>(17.86983, 4.732909, 0.637877)<br>(19.618513, 8.06484, 2.529125)<br>(15.251101, 8.397803, -1.307088)<br>(13.152869, 4.866555, -0.590875)<br>(11.307171, 8.277982, -2.473171)<br>(15.311105, 8.675464, 12.464334)<br>(19.254473, 8.957733, 13.57894)<br>(17.306732, 12.281599, 11.808552)<br>(10.294641, 8.865563, 8.731077)<br>(12.4464, 12.065001, 10.696185)

	(14.376913, 8.509558, 9.883732)
	(5.825541, 2.845736, 1.273515)
	(9.768909, 2.563467, 2.388121)
	(7.821168, -0.760399, 0.617733)
	(0.809076, 2.655637, -2.459742)
	(2.960835, -0.543801, -0.494634)
	(4.891349, 3.011642, -1.307088)
B	(3.533405, 6.139647, 5.286543)
	(5.317838, 6.368919, 17.12643)
	(14.55814, 5.381553, -5.904276)
	(16.342574, 5.152281, 5.935611)
	(16.020163, 11.676736, 17.155526)
	(13.692564, 11.571893, 5.340259)
	(6.534599, -0.155536, 5.964707)
	(4.207, -0.050693, -5.85056)

c-axis:

$P_s = \lim 1/V \sum q_i r_i = (q_{Fe^{2+}} r_{Fe^{2+}} + q_{Fe^{3+}} r_{Fe^{3+}} + q_{Co^{3+}} r_{Co^{3+}} + q_C r_C + q_N r_N + q_B r_B) / V$

$= [(2 \times e \times 10.035927) + (3 \times e \times 35.089932) + (3 \times e \times 22.444307) + (-0.5 \times e \times 134.755371) + (-0.5 \times e \times 134.55841) + (-1 \times e \times 45.05424)] \times 10^{-10}\ m / 5288.85 \times 10^{-30}\ m^3$

$= 2.451\ \mu C\ cm^{-2}$

$|P_s| = 2.451\ \mu C\ cm^{-2}$

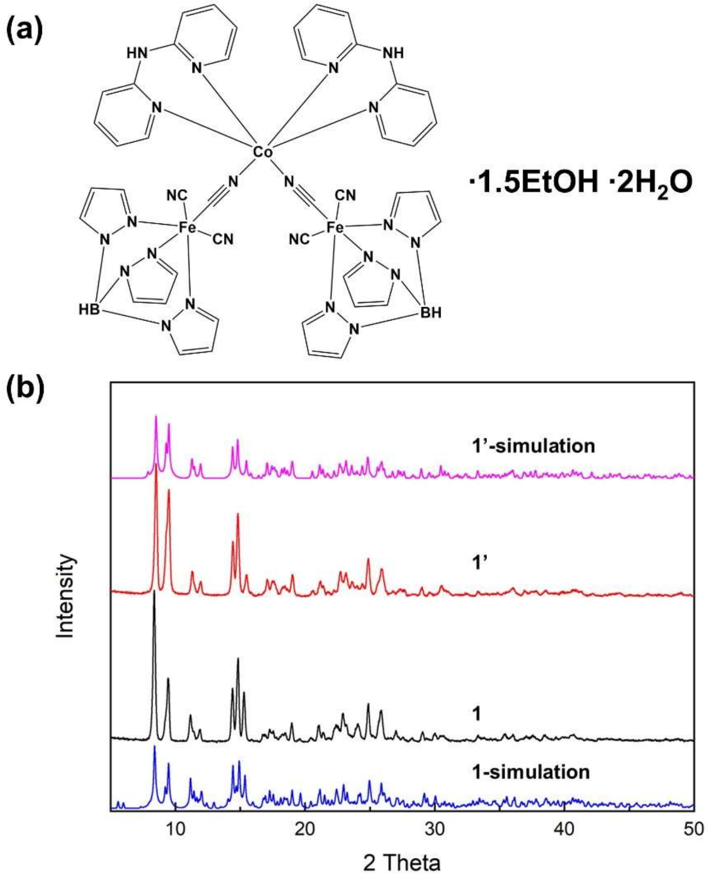

Figure S1. Molecular structure and powder X-ray diffraction (XRD) analysis. (a) Molecular structure of **1**. (b) The polycrystalline XRD patterns of a microcrystalline sample of **1** and **1'** are consistent with the pattern simulated (**1-simulation** and **1'-simulation**) on the basis of the crystal structures at 300 K, which confirmed the purity of the microcrystalline samples. The scan rate was set to 1°/min.

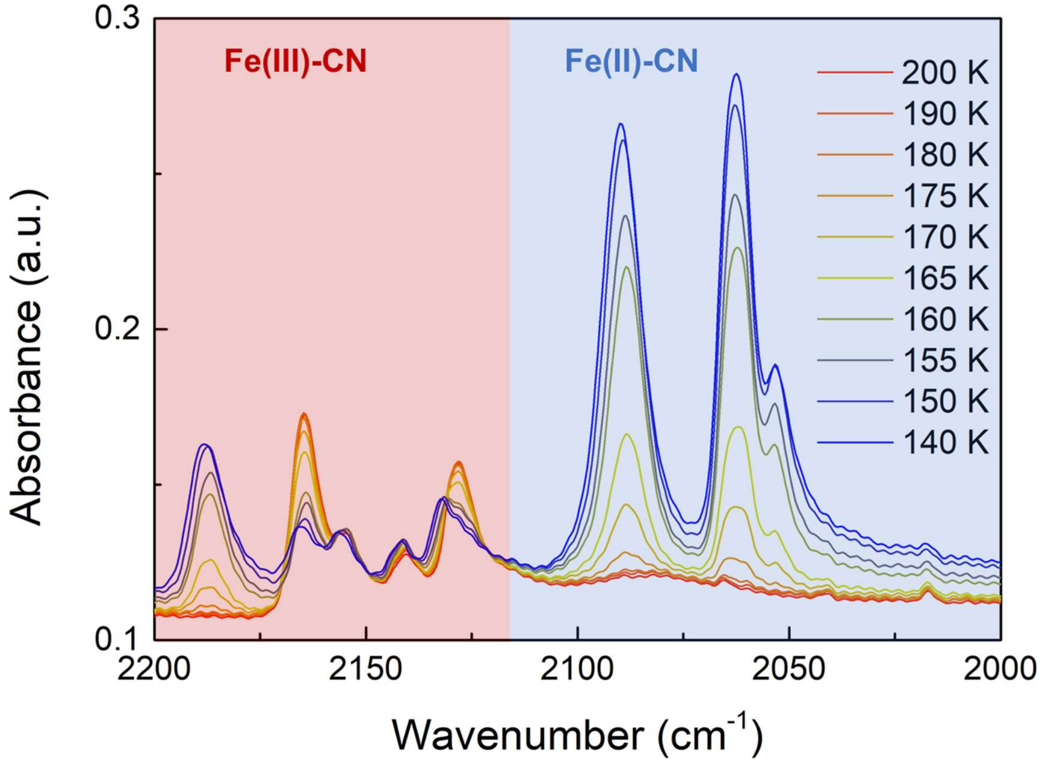

Figure S2. Temperature-dependent infrared spectra of **1**. Cyanide stretching vibration bands in the range of 2000–2200 cm^{-1} between 140 and 200 K.

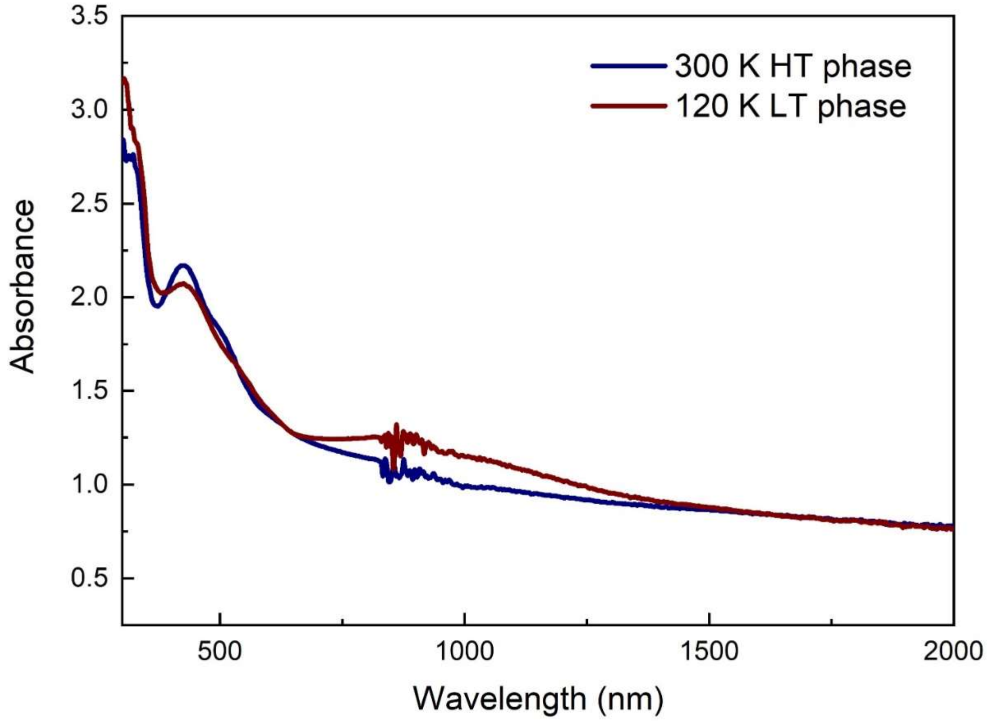

Figure S3. Temperature-dependent UV/vis absorption spectra of **1**.

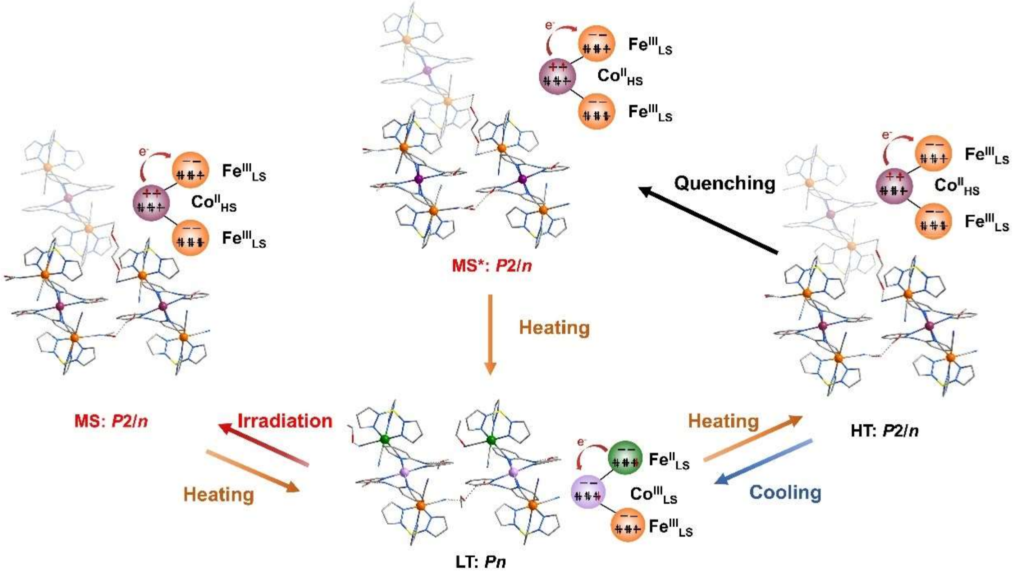

Figure S4. Molecular structure, spin and valence states of metal centers, and space group switching of **1**. The LT phase was in the polar space group *Pn*, whereas the HT, MS and MS* phases were in the nonpolar space group *P*2/*n*. The MS* phase was obtained by placing the high temperature phase directly at the low temperature, and no ETCST behavior occurred. With a temperature increase, the MS* phase returned to the LT phase via relaxation. The MS phase was obtained by irradiating LT phase by 785 nm light. During the thermal-induced ETCST process from the HT to LT phases, the orientation of part of the EtOH molecules changed from disorder to order. The gray dashed lines represent hydrogen bonds. The electron transfer process is indicated with a curved arrow. The blurred part is the adjacent structure with a disordered EtOH molecule connected by hydrogen bonds. Orange, Fe^{III}; green, Fe^{II}; lavender, Co^{III}; plum, Co^{II}; yellow, B; gray, C; red, O; blue, N; light gray, H.

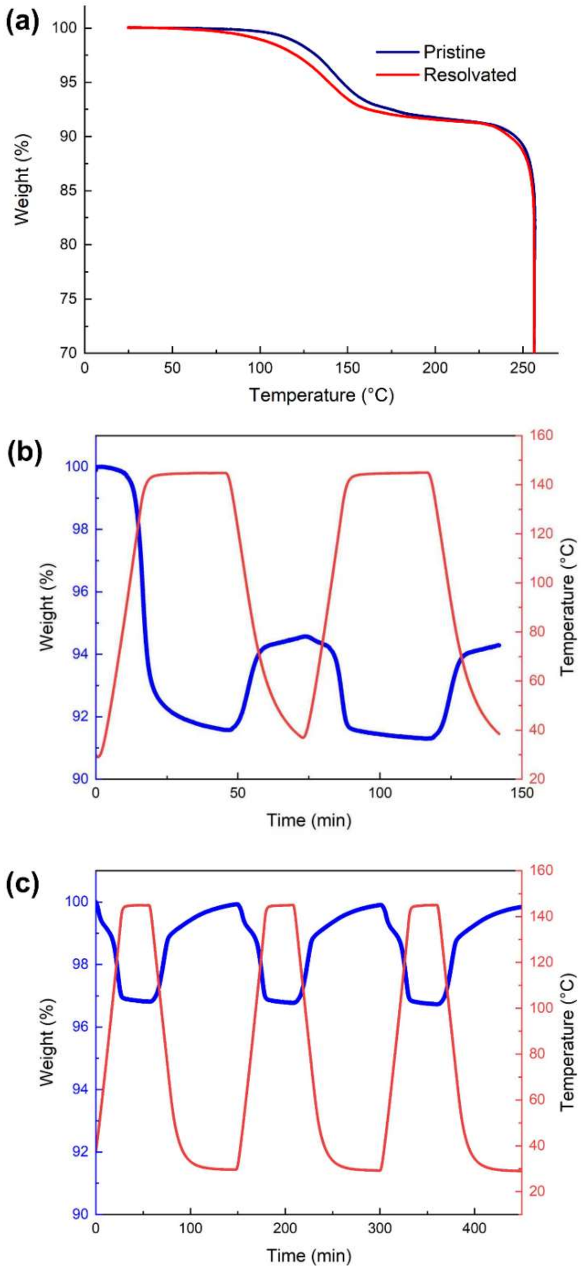

Figure S5. Thermogravimetric analyses (TGA) of **1** and **1’** under air flow. (a) TGA of a freshly prepared and resolvated samples **1**. The resolvated sample was obtained by exposing the desolvated compound **1’** to ethanol vapor for one week. Fresh sample, blue line; resolvated sample, red line. (b) TGA of a freshly prepared samples **1** for two heating and cooling cycles up to 150 °C. A new EtOH-removed phase (**1’**) corresponding to the release of 1.5 EtOH molecules is clearly observed. (c) TGA of the dihydrate **1’** for three heating and cooling cycles, suggesting the stability of this new phase.

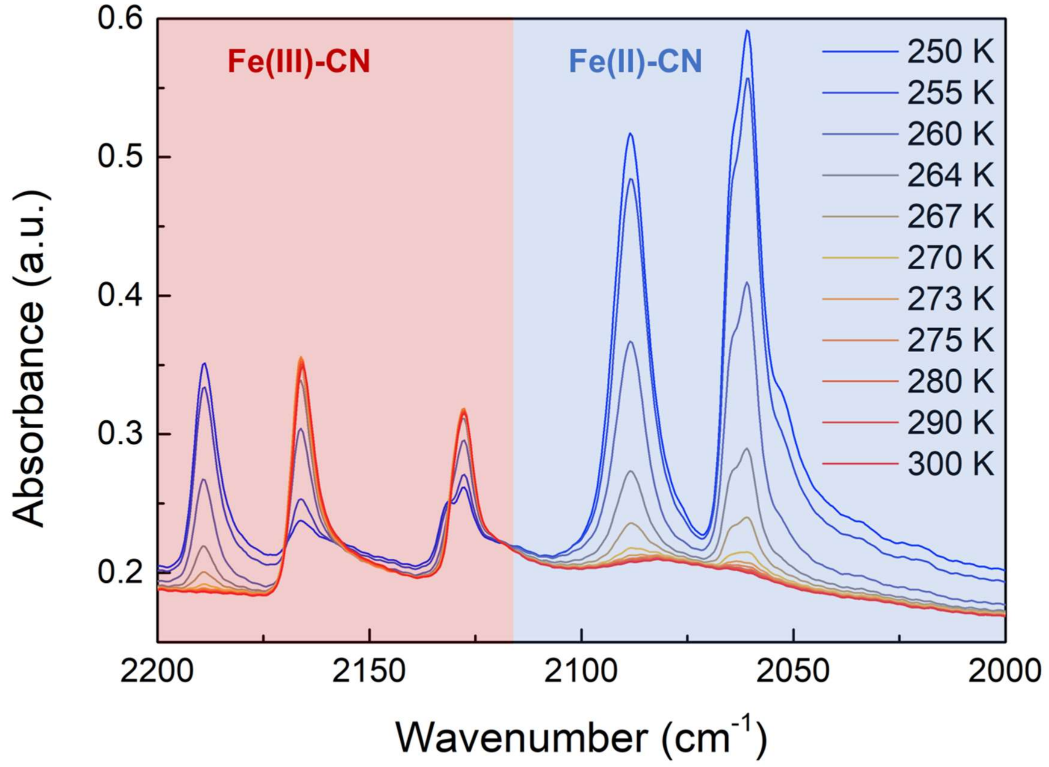

Figure S6. Temperature-dependent infrared spectra of **1'**. Cyanide stretching vibration bands in the range from 2000 to 2200 cm^{-1} between 250 and 300 K.

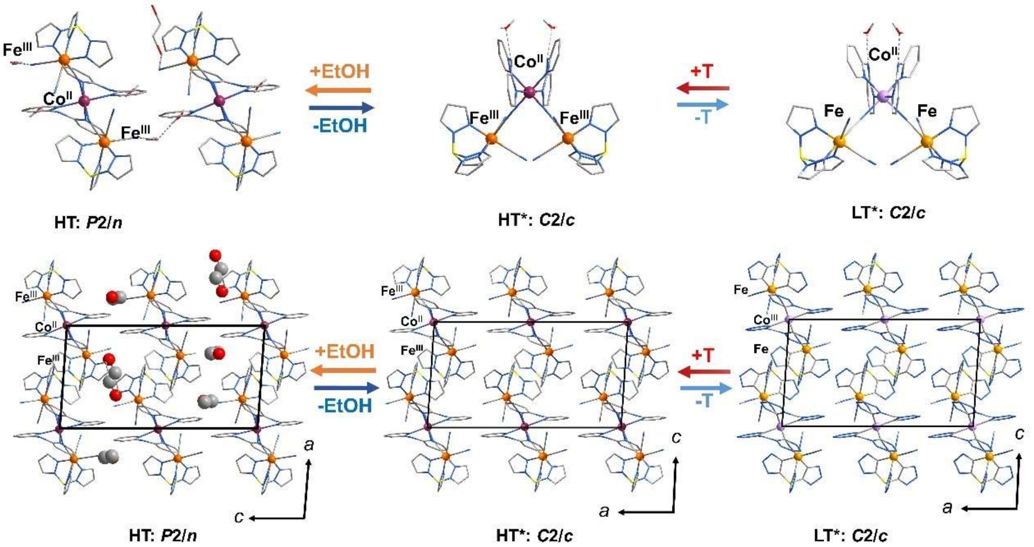

Figure S7. Molecular structure, space group, and spin and valence states of the metal centers between **1** and **1'** during dynamic vapor sorption. Top: The HT phase of **1** was in the nonpolar space group *P*2/*n*, and the LT* and HT* phases of [Fe_2Co] were in the nonpolar space group *C*2/*c*. In the LT* phase, the two irons were in the average state of $Fe^{II}{}_{LS}$ and $Fe^{III}{}_{LS}$. The transition between the LT* and HT* phases was a temperature-dependent reversible process. Bottom: Schematic representation of [Fe_2Co] in the ferroelectricity "off" state due to nondirectional ETCST. Both the LT* and HT* phases were in the nonpolar space group *C*2/*c*. Orange, Fe^{III}; green, Fe^{II}; lime, Fe; gold, Co^{II}; plum, Co^{III}; yellow, B; gray, C; red, O; blue, N; light gray, H.

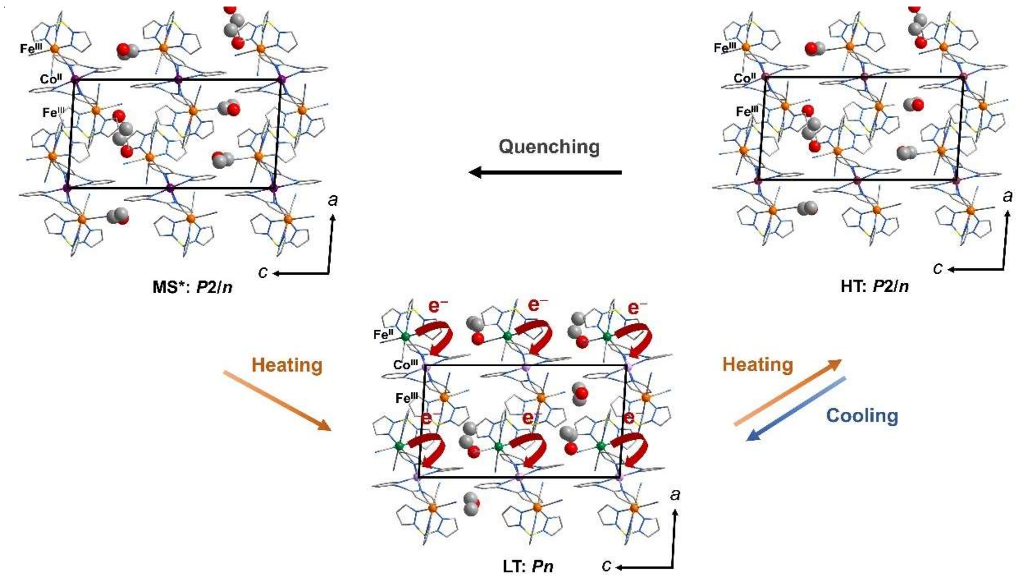

Figure S8. Molecular stacking and space group of **1** at LT, MS* and HT phase. The **1** crystalizes in a polar space group *Pn* in the LT phase, while the **1** crystalizes in the nonpolar space group *P*2/*n* in the HT phase and MS* phase. During thermo-induced ETCST process, the disordered part of ethanol molecules in the HT phase become ordered in the LT phase. Orange, Fe^{III}; green, Fe^{II}; lavender, Co^{II}; plum, Co^{III}; yellow, B; gray, C; red, O; blue, N.

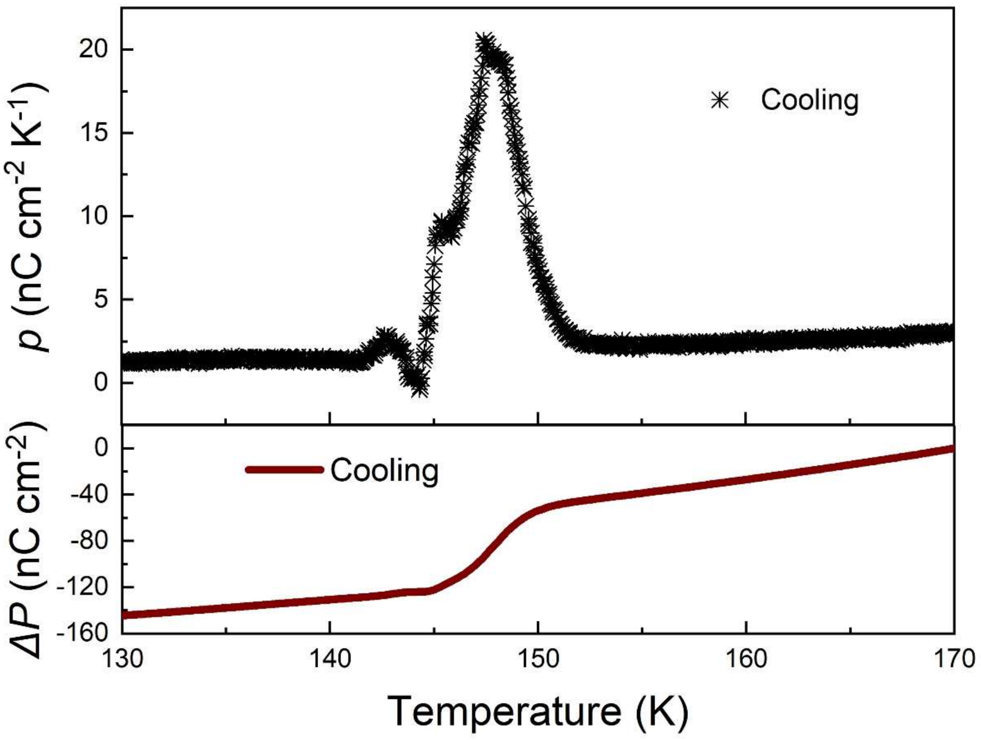

Figure S9. Temperature-dependence of pyroelectric coefficient (p) and polarization change (ΔP) based on single-crystal sample of **1**.

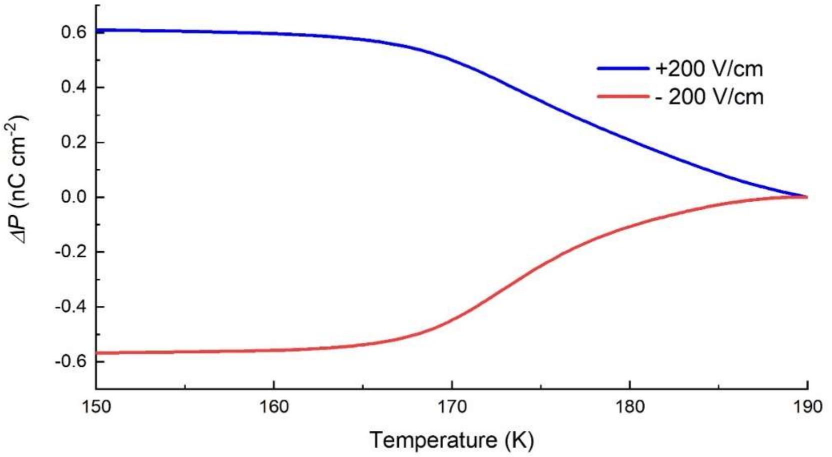

Figure S10. Temperature-dependent polarization changes based on powder compacts of **1**.

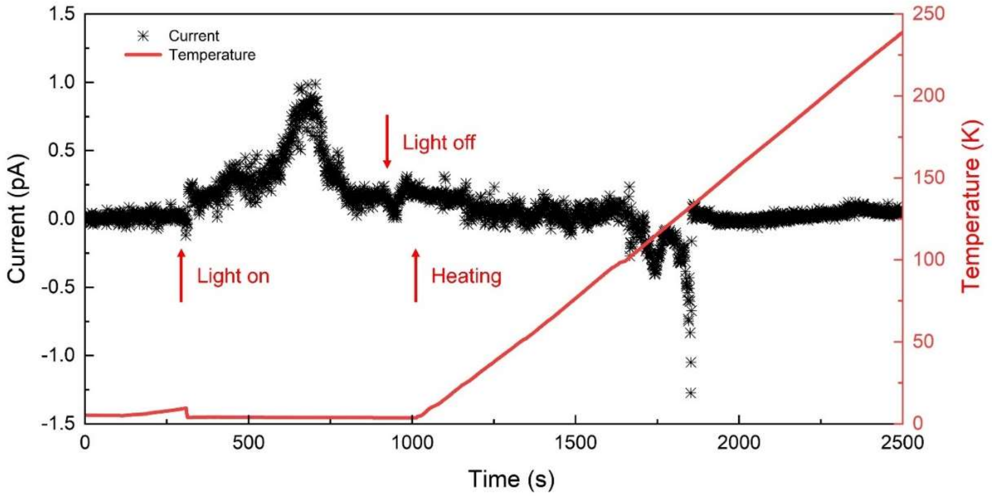

Figure S11. Raw data of pyroelectric measurements based on single-crystal sample of **1** after irradiation with 785 nm light in a sweep rate of 10 K min^{-1}.

Table S1. Crystallographic parameters of complex **1** at 100 K (LT phase), and 300 K (HT phase).

Temperature	**100 K**	**300 K**
Formula	$C_{94}H_{102}B_4Co_2Fe_4N_{48}O_7$	$C_{47}H_{51}B_2CoFe_2N_{24}O_{3.5}$
Fw	2400.72	1200.36
Space group	*Pn*	*P*2/*n*
a = (Å)	20.5103(3)	21.2823(6)
b = (Å)	11.5212(2)	12.0737(3)
c = (Å)	22.4345(4)	22.3748(5)
β = (°)	93.9340(10)	93.779(2)
V (Å^3)	5288.85(14)	5736.9(2)
Z	2	4
D_{cald}(g/cm^3)	1.508	1.390
R_{int}	0.0472	0.0277
Parameters	1452	742
F(000)	2472.0	2472.0
Reflections	122625	41978
R_1, $wR(I > 2\sigma(I))$[a]	0.0525, 0.1186	0.0429, 0.1057
R_1, wR(all data)[b]	0.0832, 0.1358	0.0694, 0.1159
Goodness-of-fit-on F^2	1.065	1.041
Flack parameter	0.063(5)	/

[a] $R = \sum||F_0|-|F_c||/\sum|F_0|$ [b] $wR = |\sum w(F_0-F_c)^2/\sum w(F_0^2)^2|^{1/2}$

Table S2. Characteristic bond lengths (Å) of complex **1** at 100 K (LT phase), 300 K (HT phase).

Temperature	**100 K**	**300 K**
Co-N_{dpa}	**Co1:** 1.936(6), 1.946(6), 1.949(6), 1.961(5)<br>**Co2:** 1.931(6), 1.934(6), 1.950(6), 1.952(6)	**Co1:** 2.1305(18), 2.1545(17)<br>**Co2:** 2.1187(18), 2.1668(17)
Co-N_{CN}	**Co1:** 1.893(7), 1.910(7)<br>**Co2:** 1.868(6), 1.910(5)	**Co1:** 2.1025(19)<br>**Co2:** 2.1052(19)
Fe-N	**Fe1:** 2.005(6), 1.998(6), 2.007(6)<br>**Fe2:** 1.993(7), 1.956(6), 1.978(6)<br>**Fe3:** 1.975(6), 1.961(6), 1.980(6)<br>**Fe4:** 2.005(5), 2.003(6), 2.009(6)	**Fe1**: 1.9681(19), 1.981(2), 1.990(2)<br>**Fe2**: 1.9766(18), 1.980(2), 1.9862(19)
Fe-C	**Fe1:** 1.850(7), 1.907(7), 1.911(8)<br>**Fe2:** 1.937(8), 1.910(10), 1.905(8)<br>**Fe3:** 1.908(7), 1.940(7), 1.941(7)<br>**Fe4:** 1.869(7), 1.911(8), 1.910(7)	**Fe1**: 1.909(2), 1.910(3), 1.935(2)<br>**Fe2**: 1.920 (2), 1.922(3), 1.929(3)

Table S3. Crystallographic parameters of complex **1** at 30 K (MS* phase), and 30 K (MS phase).

Phase	**[Fe_2Co]·1.5EtOH-MS**	**[Fe_2Co]·1.5EtOH-MS***
Formula	$C_{47}H_{51}B_2CoFe_2N_{24}O_{3.5}$	$C_{47}H_{51}B_2CoFe_2N_{24}O_{3.5}$
Fw	1200.36	1200.36
Space group	*P*2/*n*	*P*2/*n*
a = (Å)	20.9572(10)	20.9185(7)
b = (Å)	12.0232(6)	11.9704(3)
c = (Å)	22.0948(7)	22.0197(5)
β = (°)	94.315(4)	94.269(3)
V (Å^3)	5551.5(4)	5498.5(3)
Z	4	4
D_{cald}(g/cm^3)	1.436	1.450
R_{int}	0.1117	0.0887
Parameters	752	744
F(000)	2456.0	2472.0
Reflections	52754	53851
R_1, $wR(I > 2\sigma(I))$[a]	0.0727, 0.1739	0.0481, 0.1146
R_1, *wR*(all data)[b]	0.1224, 0.2049	0.0776, 0.1251
Goodness-of-fit-on F^2	1.072	1.058

[a] $R = \sum||F_0|-|F_c||/\sum|F_0|$ [b] $wR = |\sum w(F_0-F_c)^2/\sum w(F_0{}^2)^2|^{1/2}$

Table S4. Characteristic bond lengths (Å) of complex **1** at 30 K (MS* phase), and 30 K (MS phase).

Phase	**[Fe_2Co]·1.5EtOH-MS**	**[Fe_2Co]·1.5EtOH-MS***
Co-N_{dpa}	**Co1:** 2.107(4), 2.159(3)<br>**Co2:** 2.126(4), 2.145(4)	**Co1:** 2.118(2), 2.137(2)<br>**Co2:** 2.099(2), 2.149(2)
Co-N_{CN}	**Co1:** 2.096(4)<br>**Co2:** 2.074(4)	**Co1:** 2.066(2)<br>**Co2:** 2.087(2)
Fe-N	**Fe1**: 1.978(3), 1.972(4), 1.979(4)<br>**Fe2**: 1.980(4), 1.976(4), 1.953(4)	**Fe1**: 1.980(2), 1.974(2), 1.945(2)<br>**Fe3**: 1.976(2), 1.9647(19), 1.972(2)
Fe-C	**Fe1**:1.927(5), 1.918(5), 1.924(4)<br>**Fe2**:1.923(5), 1.902(4), 1.932(4)	**Fe1**:1.923(2), 1.903(3), 1.910(3)<br>**Fe3**:1.923(3), 1.910(3), 1.921(3)

Table S5. Crystallographic parameters of complex **1'** at 120 K (LT phase), and 300 K (HT phase).

Temperature	**120 K**	**300 K**
Formula	$C_{22}H_{21}BCo_{0.5}FeN_{12}O$	$C_{22}H_{21}BCo_{0.5}FeN_{12}O$
Fw	565.63	565.63
Space group	*C*2/*c*	*C*2/*c*
a = (Å)	20.1208(7)	20.8106(13)
b = (Å)	11.4493(4)	11.9718(7)
c = (Å)	22.5687(7)	22.4235(12)
β = (°)	94.392(3)	93.254(5)
V (Å^3)	5183.8(3)	5577.6(6)
Z	8	8
D_{cald}(g/cm^3)	1.450	1.347
R_{int}	0.0252	0.0487
Parameters	345	345
F(000)	2316.0	2316.0
Reflections	18623	15656
R_1, $wR(I > 2\sigma(I))^a$	0.0474, 0.1041	0.0507, 0.1261
R_1, wR(all data)^b	0.0645, 0.1119	0.0930, 0.1544
Goodness-of-fit-on F^2	1.196	1.020

$^a R = \sum||F_0|-|F_c||/\sum|F_0|$ $^b wR = |\sum w(F_0-F_c)^2/\sum w(F_0{}^2)^2|^{1/2}$

Table S6. Characteristic bond lengths (Å) of complex **1'** at 120 K (LT phase), and 300 K (HT phase).

Temperature	**120 K**	**300 K**
Co-N_{dpa}	**Co1:** 1.945(2), 1.938(2)	**Co1:** 2.104(3), 2.152(3)
Co-N_{CN}	**Co1:** 1.887(2)	**Co1:** 2.084(3)
Fe-N	**Fe1:** 1.999(2), 1.992(2), 1.987(2)	**Fe1**: 1.992(3), 1.975(3), 1.986(3)
Fe-C	**Fe1:** 1.924(3), 1.875(3), 1.925(3)	**Fe1**:1.909(3), 1.928(4), 1.935(4)

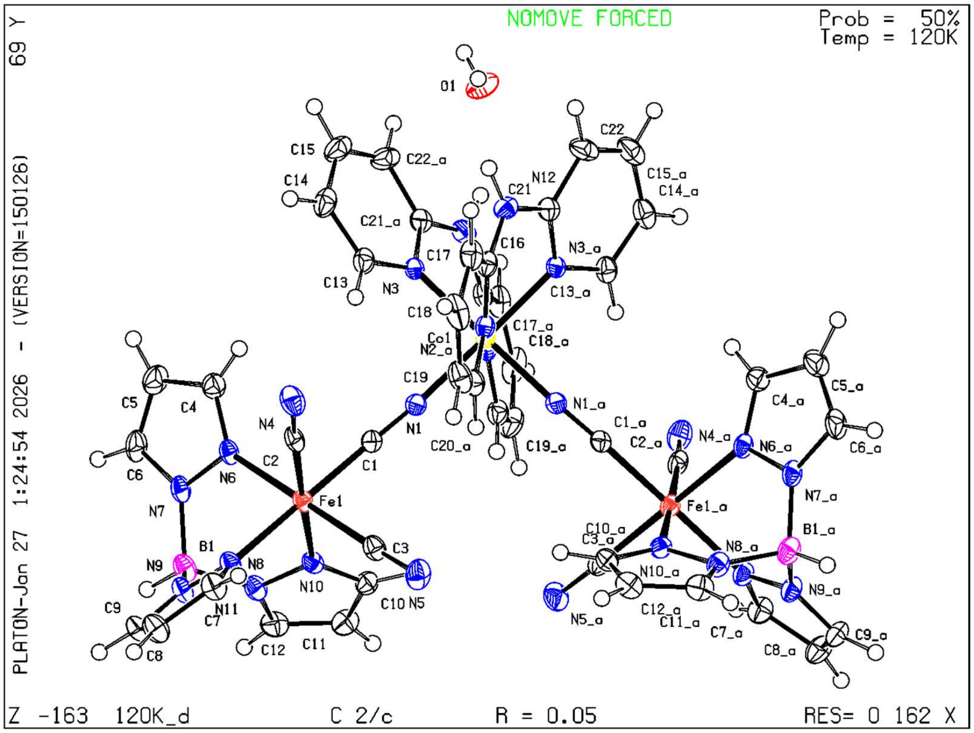

ORTEP-style illustration of LT* (CCDC 2352581)

Alert level B

PLAT601_ALERT_2_B Unit-Cell Contains Solvent Accessible VOIDS .LE. 128 Ang**3

Author Response: Voids caused by desorption

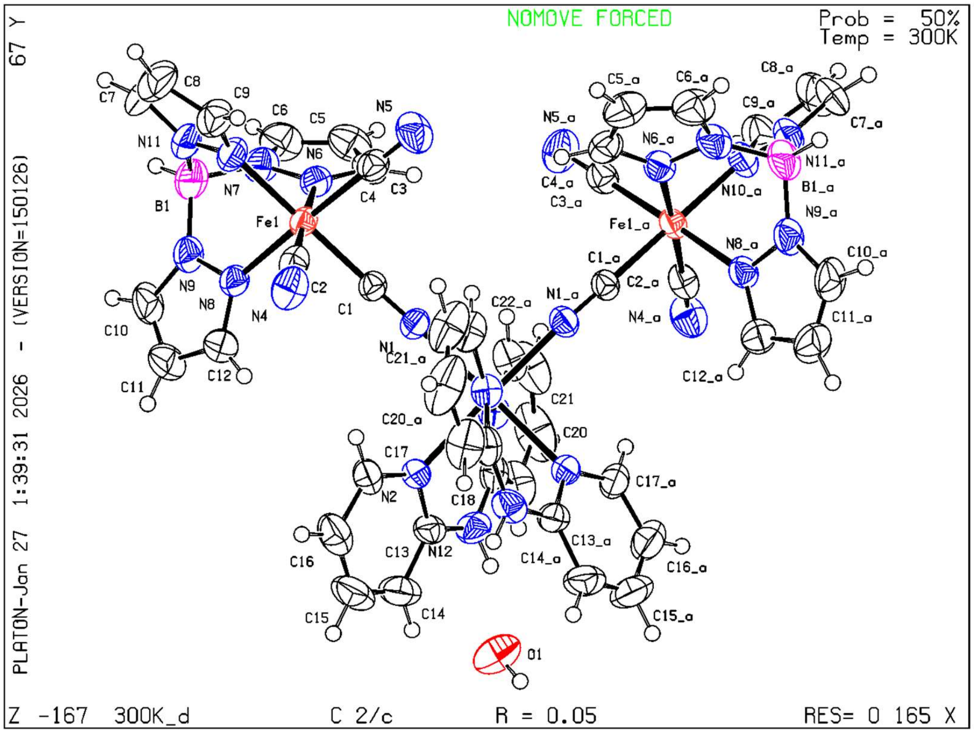

ORTEP-style illustration of HT* (CCDC 2352582)

Alert level B

PLAT029_ALERT_3_B _diffrn_measured_fraction_theta_full value Low . 0.953 Why?

Author Response: the crystal badly diffracting after desorption

PLAT601_ALERT_2_B Unit-Cell Contains Solvent Accessible VOIDS .LE. 185 Ang**3

Author Response: Voids caused by desorption

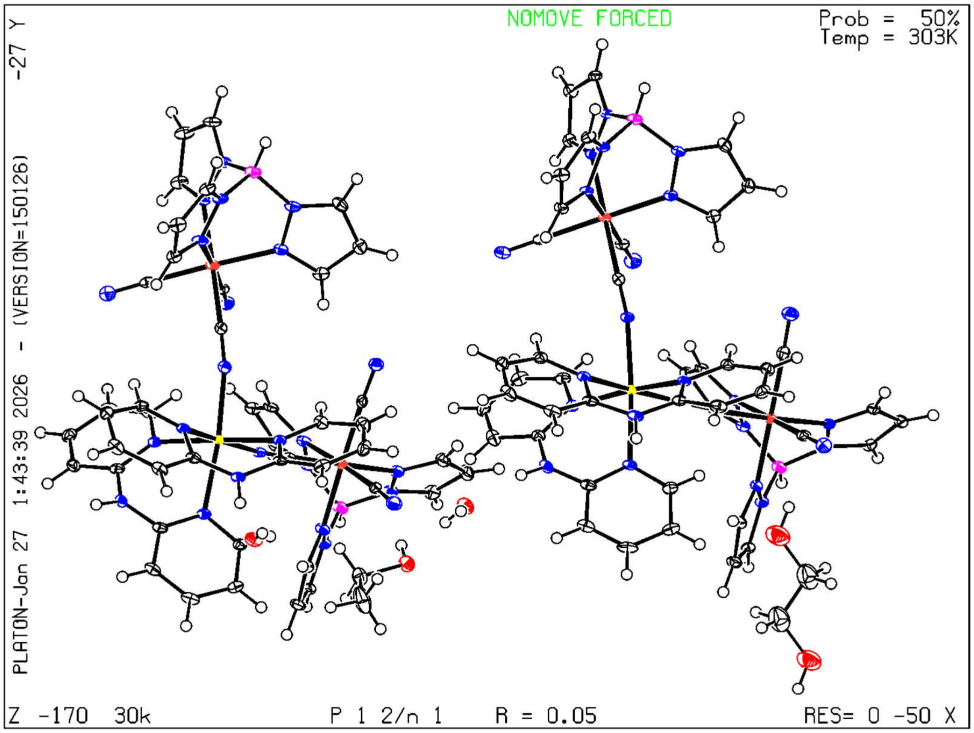

ORTEP-style illustration of MS* (CCDC 2352583)

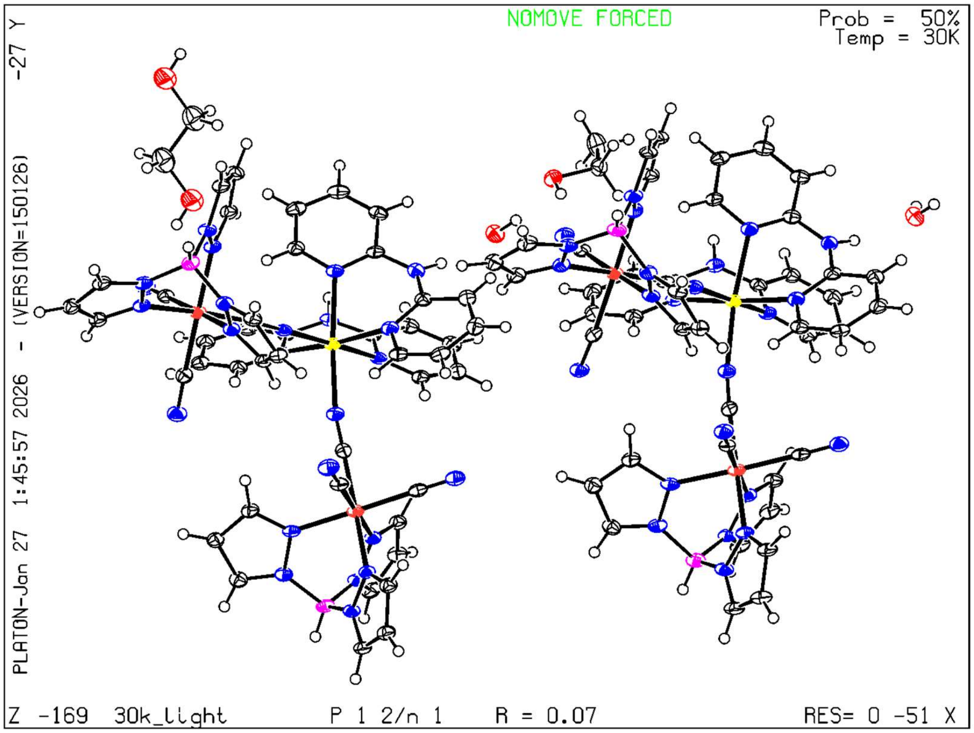

ORTEP-style illustration of MS (CCDC 2352585)

Alert level B

PLAT973_ALERT_2_B Check Calcd Positive Resid. Density on Co1 1.85 eA-3

Author Response: the crystal badly diffracting after light irradiation

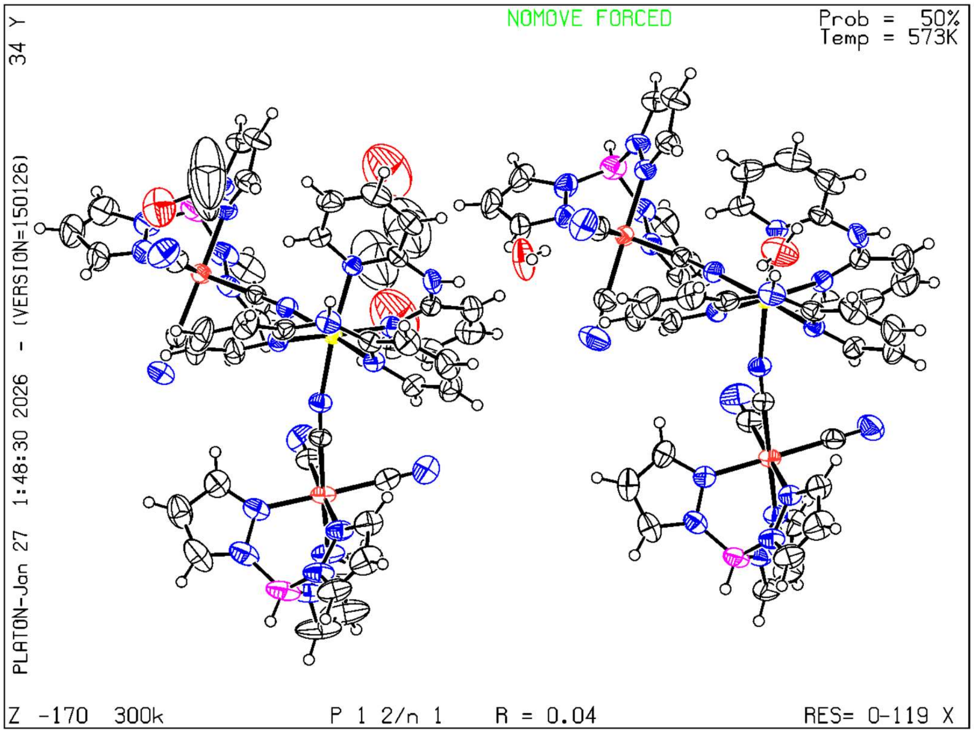

ORTEP-style illustration of HT (CCDC 2352586)

Alert level B

PLAT315_ALERT_2_B Singly Bonded Carbon Detected (H-atoms Missing). C46 Check

Author Response: It's hard to locate H atoms due to severe disorder of ethanol.

PLAT417_ALERT_2_B Short Inter D-H..H-D H2A ..H3 . 2.02 Ang.

3/2-x,1+y,1/2-z = 2_665 Check

Author Response: Multiple orientations lead to H-H contacts due to disordered ethanol.

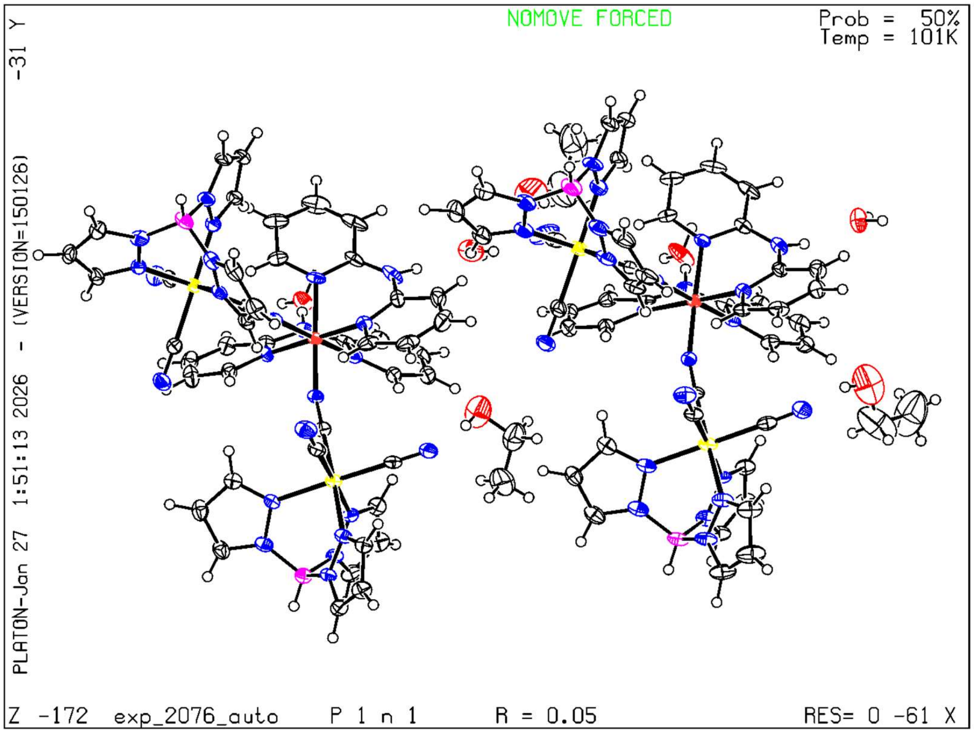

ORTEP-style illustration of LT (CCDC 2377474)

Alert level B

PLAT987_ALERT_1_B The Flack x is >> 0 - Do a BASF/TWIN Refinement Please Check

Author Response: domains are formed during the ferroelectric phase transition.
